# Cellular and Animal Model Studies on the Growth Inhibitory Effects of Polyamine Analogues on Breast Cancer

**DOI:** 10.3390/medsci6010024

**Published:** 2018-03-13

**Authors:** T. J. Thomas, Thresia Thomas

**Affiliations:** 1 Department of Medicine, Rutgers Robert Wood Johnson Medical School and Rutgers Cancer Institute of New Jersey, Rutgers, The State University of New Jersey, 675 Hoes Lane West, KTL Room N102, Piscataway, NJ 08854, USA; 2 Retired from Department of Environmental and Occupational Medicine, Rutgers Robert Wood Johnson Medical School and Rutgers Cancer Institute of New Jersey, Rutgers, The State University of New Jersey, 675 Hoes Lane West, Piscataway, NJ 08854, USA; thomasthresia@gmail.com

**Keywords:** polyamines, ornithine decarboxylase, polyamine analogs, spermidine/spermine *N*^1^-acetyl transferase, spermine oxidase, bis(ethyl)polyamine analogs, breast cancer, MCF-7 cells, transgenic mice

## Abstract

Polyamine levels are elevated in breast tumors compared to those of adjacent normal tissues. The female sex hormone, estrogen is implicated in the origin and progression of breast cancer. Estrogens stimulate and antiestrogens suppress the expression of polyamine biosynthetic enzyme, ornithine decarboxylate (ODC). Using several bis(ethyl)spermine analogues, we found that these analogues inhibited the proliferation of estrogen receptor-positive and estrogen receptor negative breast cancer cells in culture. There was structure-activity relationship in the efficacy of these compounds in suppressing cell growth. The activity of ODC was inhibited by these compounds, whereas the activity of the catabolizing enzyme, spermidine/spermine *N*^1^-acetyl transferase (SSAT) was increased by 6-fold by bis(ethyl)norspermine in MCF-7 cells. In a transgenic mouse model of breast cancer, bis(ethyl)norspermine reduced the formation and growth of spontaneous mammary tumor. Recent studies indicate that induction of polyamine catabolic enzymes SSAT and spermine oxidase (SMO) play key roles in the anti-proliferative and apoptotic effects of polyamine analogues and their combinations with chemotherapeutic agents such as 5-fluorouracil (5-FU) and paclitaxel. Thus, polyamine catabolic enzymes might be important therapeutic targets and markers of sensitivity in utilizing polyamine analogues in combination with other therapeutic agents.

## 1. Introduction

Breast cancer is a major public health problem and is the most common cancer in women worldwide, with nearly 1.7 million new cases diagnosed in 2012 (second most common cancer overall) [1]. For women in the United States of America in 2017, the estimation is 252,710 new cases of invasive breast cancer, 63,410 new cases of breast carcinoma in situ and 40,610 breast cancer deaths per year [2]. Although breast cancer incidence rates are highest in non-Hispanic white women, breast cancer death rates are highest in African American women. Breast cancer is a heterogeneous disease and harbors different receptors and in some cases no recognized receptor driving the disease [3]. The female hormone, estrogen is implicated in the origin and progression of breast cancer and approximately 70% of breast tumors harbor the receptor protein, estrogen receptor (ER) [4,5,6]. There are two forms of ER, ERα and ERβ, with several isoforms of each of these subtypes [6,7]. Binding of estrogen with ERα provokes conformational changes in ERα, enabling it to recruit coactivator proteins and recognize the estrogen response element (ERE) in responsive genes to facilitate breast cancer growth. ERα-positive tumors are responsive to antiestrogens, such as tamoxifen [8]. Aromatase inhibitors are also available to treat these tumors by blocking the synthesis of estrogens [9]. Approximately 15% of breast tumors harbor the human epidermal receptor-2 (HER-2) and these tumors respond to a monoclonal antibody, Herceptin, targeted to HER-2 [10]. A subset of 15–20% of breast tumors lacks ER, HER-2 and progesterone receptor (PR) and this subtype is classified as triple negative breast cancer (TNBC). There is no targeted therapy for TNBCs [11]. In the case of ERα- and HER-2 positive breast tumors, drug resistance develops in the course of therapy and hence new drugs are needed for treating all forms of breast cancer [12]. Research on polyamine analogues have been undertaken in this context [13,14,15,16,17].

## 2. Polyamine Metabolism and Breast Cancer

Natural polyamines (putrescine, spermidine and spermine) are ubiquitous cellular cations and play important roles in cell growth and differentiation [18,19]. Polyamine levels are elevated in cancer cells compared to that in adjacent tissues [14,20,21]. Rise in cellular polyamine levels, associated with up-regulation of polyamine biosynthetic enzymes, is characteristic of increased proliferation of cancer cells. Polyamines are positively charged at physiological pH and ionic conditions and hence they can interact with a variety of biological targets, including membrane phospholipids, proteins and nucleic acids through electrostatic interactions with negatively charged groups on these macromolecules [22,23,24,25]. Polyamines are capable of facilitating the interaction of transcription factors—such as ERα and nuclear factor kappa B (NF-κB)—with their response elements in breast cancer [26,27,28]. In addition, polyamines enhanced the bending of estrogen response element in the presence of ERα [29]. 

**Figure 1:**
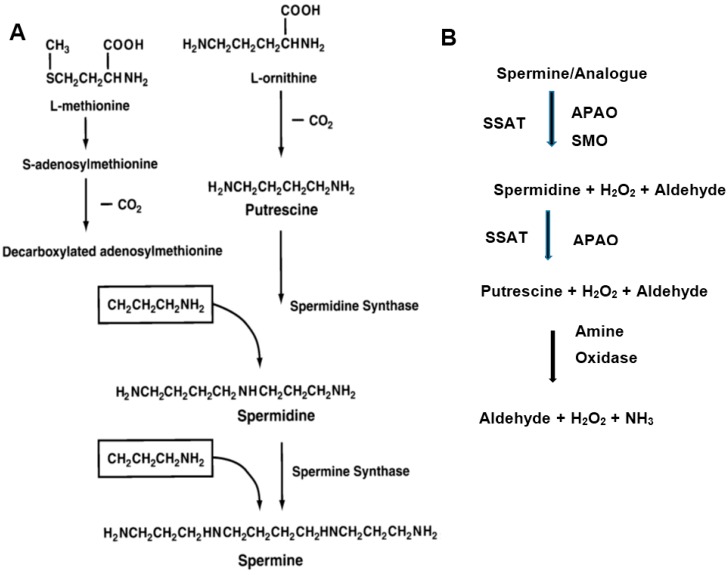
Schematic representation of the polyamine biosynthetic (**A**) and catabolic (**B**) pathways. (**A**) Putrescine is formed by the decarboxylation of ornithine by ornithine decarboxylate (ODC). Spermidine is formed by the action of spermidine synthase that links putrescine to an aminopropyl group derived from decarboxylated S-adenosylmethionine, a reaction product of AdoMetDC. Spermine is synthesized from spermidine by a similar process by spermine synthase; (**B**) Spermine and spermidine are first acetylated spermidine/spermine *N*^1^-acetyltransferase (SSAT) and then oxidized by *N*^1^-acetylpolyamine oxidase (APAO). Spermine oxidase (SMO) degrades unmodified spermine/internalized analogue. H_2_O_2_ and 3-aceto-aminopropanal are among the degradation products [17]. Multiple amine oxidases are also involved in the degradation of diamines and polyamines, producing H_2_O_2_ and aldehydes [33].

Cellular polyamine levels are exquisitely regulated by biosynthetic and catabolic enzymes (Figure 1) [17,18,19]. The biosynthetic enzymes are ornithine decarboxylase (ODC), adenosylmethionine decarboxylase (AdoMetDC) and aminopropyl transferase (spermidine synthase and spermine synthase). Increased ODC activity in human breast cancer tissues was found to be an independent adverse prognostic factor for recurrence and death. The catabolic enzymes are spermidine/spermine *N*^1^-acetyltransferase (SSAT), *N*^1^-acetylpolyamine oxidase (APAO) and spermine oxidase (SMO) [30,31,32]. Induction of SMO and the concomitant production of reactive oxygen species (ROS) have been linked to inflammation-associated cancers [32]. Multiple amine oxidases are also implicated in the degradation of diamines and polyamines, resulting in the production of H_2_O_2_ and aldehydes [33]. In addition, polyamine transport systems control the cellular import and export of polyamines to maintain polyamine homeostasis [34]. Antizyme proteins further control polyamine levels by binding to ODC and facilitating its degradation and thereby regulating polyamine pools in the cell [35]. The polyamine metabolic and transport pathways have been attractive targets for therapeutic intervention in cancers, including breast cancer [14,18,20,21,36,37]. 

Estradiol stimulates ODC at the mRNA, enzyme activity and polyamine biosynthesis levels and ODC knockout diminishes the mRNA and protein expression of ERα in ERα-positive MCF-7 and T-47D human breast cancer cells [38,39]. Inhibition of ODC by α-difluoromethylornithine (DFMO), an irreversible inhibitor of the enzyme, blocked the growth promoting activity of estradiol in *N*-methyl-*N*-nitrosourea-induced Sprague-Dawley rat mammary tumor grown in the soft agar clonogenic assay as well as xenograft growth in animals [40]. This drug was once considered as a promising anticancer drug, interfering with polyamine pathway and it was evaluated in Phase I/II clinical trials as a single agent as well as in combination treatment protocols [14]. However, this enzyme inhibitor exerted only limited therapeutic effects in breast and other cancers, probably due to the high concentrations required to inhibit ODC, poor cellular uptake and the versatility of polyamine pathway to replenish cellular polyamine pools. Interestingly, the use of DFMO in combination with other chemopreventive agents showed promising results in colorectal cancer [41]. AdoMetDC inhibitors also suppressed breast cancer cell growth in a structure-dependent manner [42].

Recent studies showed that endoxifen, an active metabolite of tamoxifen, suppressed the activity of ODC and AdoMetDC and induced SMO and APAO in MCF-7 cells [37]. Cellular putrescine and spermidine levels were reduced in response to endoxifen treatment. Results of this study indicated that in addition to the reduction of polyamine biosynthetic enzymes, induction of catabolic enzymes could play a role in antiproliferative effects of tamoxifen and endoxifen. Induction of SMO and APAO lead to degradation of polyamines and the production of H_2_O_2_, contributing of cell death by apoptosis [36,43,44]. Thus, the polyamine metabolic pathway plays a critical role in the mechanism of action of antiestrogens. Although most polyamine-related breast cancer studies were conducted using ERα-positive cells, polyamine biosynthetic inhibitors suppressed the proliferation of ERα-negative breast cancer cells also [45,46]. 

## 3. Polyamine Analogues and Breast Cancer Therapeutics

Despite initial success in cellular and animal models of several cancers, including breast cancer, clinical advance of DFMO and related polyamine biosynthetic inhibitors was hampered by lack of efficacy and adverse side effects [47,48]. Polyamine analogues were synthesized for cancer therapeutics on the premise that these molecules could utilize the polyamine transport pathway for cellular internalization and disrupt cellular functions of natural polyamines [13,14,15,16,17,20,49,50,51]. Early developments in this area involved the synthesis of polyamine analogues with structural alterations in the number of methylene groups between the amino and imino groups of natural polyamines. Porter and Bergeron found that homologues of putrescine and spermidine were taken up by L1210 leukemia cells and that analogues with small changes in carbon chain length could reverse DFMO-mediated cell growth inhibition, whereas large changes in chain length deprived the analogue’s ability to support cell growth [49]. A differential effect of putrescine analogues in preventing DFMO-mediated cell growth inhibition and normal immune response was also reported [52]. DFMO inhibited cell growth and suppressed putrescine and spermidine levels in MCF-7 breast cancer cells, whereas putrescine and its close homologues, diaminopropane and diaminopentane partially reversed the growth inhibitory effects of DFMO [38]. However, diaminoethane was not able to reverse DFMO’s growth inhibitory effect on MCF-7 cells. Similar results were obtained with a series of spermidine homologues of the structure, H_2_N(CH_2_)_n_NH(CH_2_)_3_NH_2_ (where *n* = 2 to 8; abbreviated as AP_n_ with *n* = 4 for spermidine). Spermidine was most effective in reversing the effects of DFMO, whereas compounds with shorter or longer methylene bridging regions were less effective. The homologue abbreviated as AP8 (*n* = 8) was ineffective in reversing the growth inhibitory effects of DFMO. In addition, AP8 inhibited DNA synthesis by 66% as a single agent, as measured by [^3^H]-thymidine incorporation assay [38]. These data suggested that certain polyamine analogues could disrupt breast cancer growth in cell culture conditions. 

**Figure 2:**
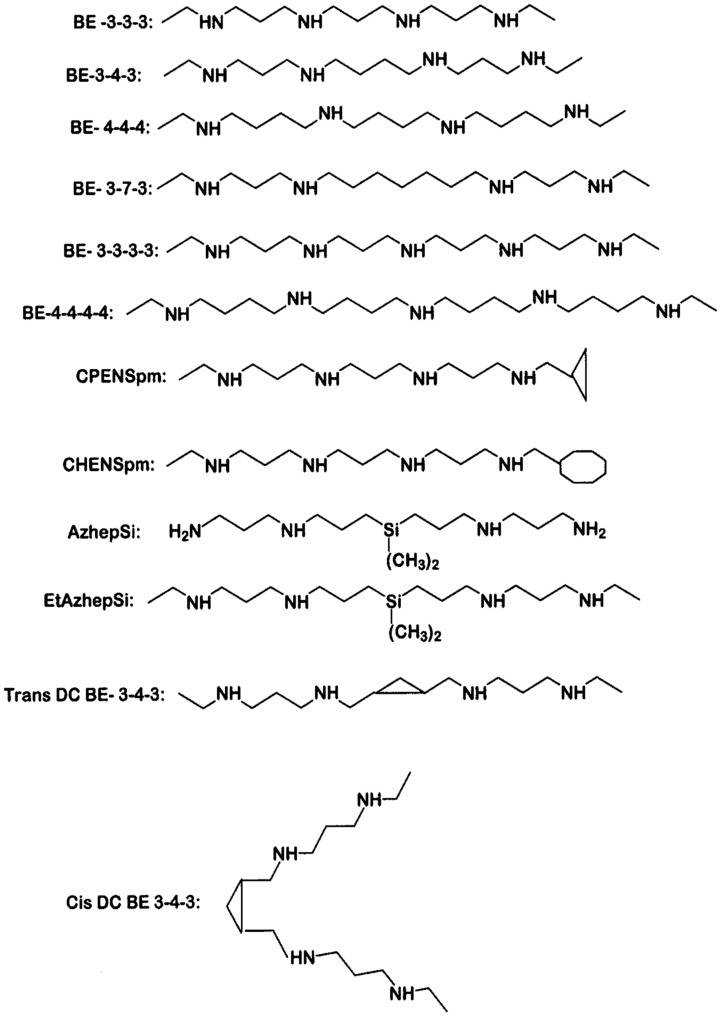
Chemical structures of polyamine analogs. Abbreviations are as follows; BE-3-3-3, *N*^1^,*N*^11^-bis(ethyl)norspermine (BENSpm or DENSpm); BE-3-4-3, *N*^1^,*N*^12^-bis(ethyl)spermine; BE-4-4-4, *N*^1^,*N*^14^-bis(ethyl)homospermine; BE-3-7-3, *N*^1^,*N*^15^-bis-[3-(ethylamino)propyl]-1-17-heptane diamine; BE-3-3-3-3, 1,15-bis(ethylamino)-4,8,12-triazapentadecane; BE-4-4-4-4, 1,19-bis(ethylamino)-5,10,15- triazanonadecane; CPENSpm, *N*^1^-ethyl-*N*^11^-(cyclopropyl)methyl-4,8-diazaundecane; CHENSpm, *N*^1^-ethyl-*N*^11^-(cycloheptyl)methyl)-4,8-diazaundecane; AzhepS1, bis(7-amino-4-azaheptyl) dimethyl- silane; EtAzhepSi, bis(7-ethylamino-4-azaheptyl)dimethylsilane; Trans DCBE-3-4-3, trans isomer of BE-3-4-3 with central 1,2, dimethylcyclopropyl residue; Cis DCBE-3-4-3, cis isomer of BE-3-4-3 with central 1,2-dimethylcyclopropyl residue.

Polyamines with terminal amino groups are good substrates for amine oxidases, including SMO and APAO [53,54]. Porter and Bergeron designed and synthesized bis(ethyl) spermine analogues (Figure 2) as cancer therapeutic agents, with the goal of exploiting the polyamine transport system to accumulate these analogues within the cancer cell, thereby downregulating polyamine biosynthesis and depleting natural polyamines [55,56,57,58]. Consequently bis(ethyl) substituted spermine and spermidine analogues were studied for their effectiveness in suppressing the growth of different cancer cell lines in culture and xenograft models [59,60,61]. Davidson et al. [62] found that bis(ethyl)spermine (BE-3-4-3) could inhibit the growth of six breast cancer cell lines, with half maximal inhibitory concentration (IC_50_) values in the micromolar range. In addition to progressive depletion of intracellular polyamines over a period of 6 days, this compound induced polyamine catabolic enzyme, SSAT by 8-12-fold in selected cell lines. 

Our laboratory conducted a detailed study of the effects of 6 bis(ethyl)spermine analogues (BE-3-4-3, BE-4-4-4, BE-3-3-3, BE-3-7-3, BE-3-3-3-3 and BE-4-4-4-4) on cell growth, activities of polyamine metabolic enzymes, intracellular polyamine levels and analogue uptake in breast cancer cells [63]. The IC_50_ values for cell growth inhibition of BE-3-4-3, BE-3-3-3 and BE-4-4-4 were in the range of 1–2 μM, whereas BE-3-7-3, BE-3-3-3-3 and BE-4-4-4-4 had IC_50_ values of ~5 μM. These values were comparable for three cell lines: ERα-positive MCF-7, HER-2-positive SK-BR-3 and triple negative MDA-MB-231 cells. Colony formation of MCF-7 cells in soft agar showed a concentration-dependent decrease in the number of colonies per well after 14 days of treatment. All compounds induced apoptosis of MCF-7 cells at 4–6 days of treatment with 10 μM drug concentration, although structural effects were evident. There was a facile transport of analogues within MCF-7 cells, although BR-3-4-3 had the highest level of transport because of its structural similarity to that of natural spermine (3-4-3). 

All six bis(ethyl)spermine analogues selected in this study inhibited ODC activity and suppressed intracellular levels of putrescine and spermidine in MCF-7 cells [63]. Spermine levels were significantly reduced by BE-3-4-3, BE-3-3-3, BE-3-3-3-3 and BE-4-4-4-4, whereas BE-4-4-4 and BE-3-7-3 had no significant effect. SSAT activity was increased by 3- to 6-fold by BE-3-4-3, BE-3-3-3 and BE-3-3-3-3, although other analogues exerted no significant effect. These results indicated that the polyamine metabolic pathway was affected by bis(ethyl)polyamine analogues in breast cancer cells. Molecular modeling studies further suggested a correlation between anti-proliferative activity of the analogues and their ability to dock into DNA major or minor grooves [63].

**Figure 3:**
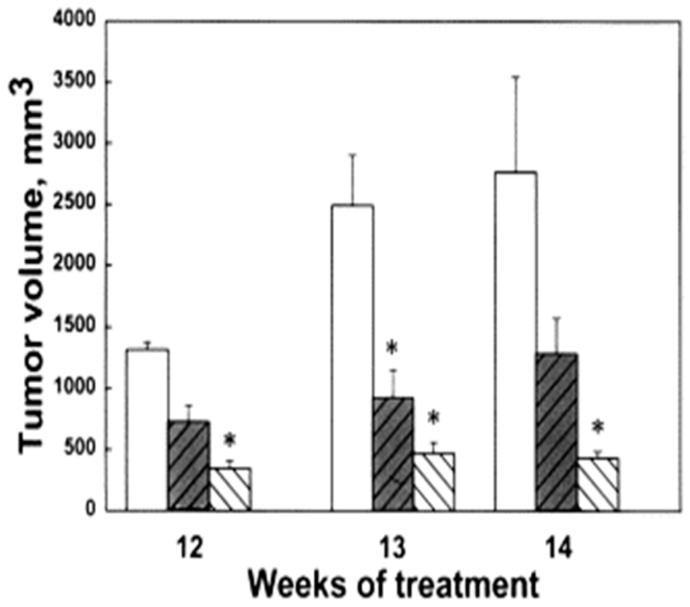
Effect of bis(ethyl)polyamines on tumor volume. The data presented are the average values determined on weeks 12, 13 and 14 of treatment of FVB/NTgN (MMTVneu) transgenic mice. Bars indicate control (unfilled bar), BE-3-3-3 treatment (darkened bar) and BE-3-3-3-3 treatment (striped bar) groups. * Statistically significant (*p* < 0.05) compared with controls, as determined by ANOVA followed by Dunnett’s test. Reproduced with permission from [66].

**Figure 4:**
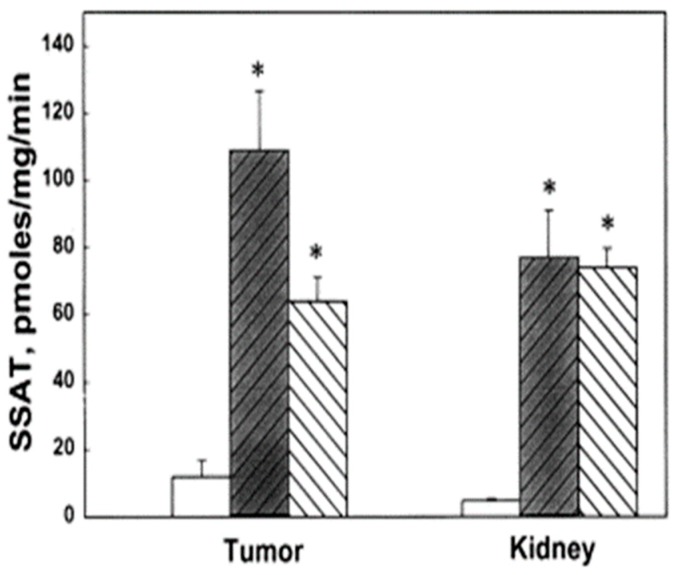
Effect of bis(ethyl)polyamines on SSAT activity in tumors and kidneys of FVB/NTgN (MMTVneu) mice. SSAT levels of control (open bar) and treatment groups, BE-3-3-3 (darkened bar) and BE-3-3-3-3 (striped bar). * Statistically significant (*p* < 0.01) compared with controls by ANOVA followed by Dunnett’s test. Reproduced with permission from [66].

We evaluated the anti-tumor effects of BE-3-3-3 and BE-3-3-3-3 using a HER-2-positive transgenic mouse model of breast cancer. Prior studies showed that increased polyamine biosynthetic activity critically interacted with HER2/neu in promoting human mammary cell transformation in culture [64]. FVB/NTgN (MMTVneu) transgenic mice developed mammary tumors at about four months of age, with a median incidence of 6.8 months [65]. Treatment of FVB/NTgN mice with BE-3-3-3 or BE-3-3-3-3 resulted in a 3- to 4-fold reduction in tumor volume compared to that of control mice (Figure 3) [66]. The activity of SSAT was determined from tumors and kidneys of control group and treatment groups. SSAT activity was significantly higher in tumors and kidneys of treatment groups than to that of controls (Figure 4). BE-3-3-3-3 was more effective than BE-3-3-3 in reducing tumor volume and inducing SSAT.

Among the group of bis(ethyl) polyamine analogues, BE-3-3-3, BE-3-4-3, BE-3-7-3 and BE-4-4-4-4, BE-3-3-3 was the most promising antitumor drug by in vitro studies. In Phase II trials, no evidence of clinical activity was detected, although this compound was reasonably tolerable [67]. Anti-proliferative action in the pre-clinical models suggested potential combination therapy approaches for this compound. Balabhadrapathruni et al. [68] showed that a combination of BE-3-3-3 and the pure antiestrogen, ICI 182780 caused down-regulation of the anti-apoptotic Bcl-2 and Bcl-XL proteins and increased the level of the pro-apoptotic Bax protein in MCF-7 and T-47D breast cancer cells. The efficacy of polyamine analogues on breast cancer cells might be governed in part by their effects on the expression of proapoptotic and antiapoptotic proteins in these cells [69]. In the case of ERα-positive tumors, the involvement of genomic and non-genomic pathways has also to be considered in mechanistic studies [70].

The activity of SMO is also affected by polyamine analogues. As shown in our recent study with endoxifen and by other studies, SMO induction is a remarkable chemotherapeutic target [36,71,72,73]. Purvalanol, a specific CDK inhibitor with apoptosis inducing activity in breast cancer cells, also induced SSAT, APAO and SMO in MCF-7 and MDA-MB-231 breast cancer cells [74]. Cervelli et al. [43] analyzed SMO mRNA and enzyme activity in breast cancer tissues and non-tumor samples. Lower levels of this enzyme were present in tumor samples than that in non-tumor tissues. Analogues BE-3-3-3 and CPENSpm were also found to be SMO inhibitors, a likely reason for the poor positive outcomes of these compounds in Phase I and Phase II clinical trials. 

Combination treatment of BE-3-3-3 with 5-FU or paclitaxel resulted in the induction of SSAT mRNA and activity in MCF-7 and MDA-MB-231 cells compared to the effect of either drug alone [75]. Spermine oxidase mRNA and activity were increased by polyamine analogues in MDA-MB-231 cells. The in vivo therapeutic efficacy of B-3-3-3 alone and in combination with paclitaxel on tumor regression was reported from studies on xenograft mice models generated with MDA-MB-231 cells [71]. Intraperitoneal exposure to BE-3-3-3 or paclitaxel singly and in combination for 4 weeks resulted in significant inhibition in tumor growth. These findings suggested that synergistic drug response was realized with combinations of polyamine analogues and chemotherapeutic agents. Nair et al. [76] found a synergistic growth inhibitory effect of 2-methoxyestradiol and BE-3-3-3 on MCF-7 cells, as determined by Chou analysis for synergism. Synergistic growth inhibitory effect was also found with BE-3-3-3 and 5-FU [71]. These studies suggest that BE-3-3-3 might be a useful drug in combination therapeutic approaches for breast cancer treatment.

Palladination of polyamines analogues was also used as an effective strategy to inhibit breast cancer cell growth [77]. In contrast to the platination of CPENSpm, which reduced cytotoxicity, palladination of BENSpm resulted in enhanced cytotoxicity, which might be due to differences in the cellular uptake of Pd-BENSpm and Pt-CPENSpm. Palladinated bisethylnorspermine (Pd-BENSpm) was the most efficient compound in the induction of DNA damage and decrease in colony formation in soft agar. Our group has also studied the effects of a bis(benzyl)spermine analogue on MCF-7 cells growing in culture and nude mice xenografts [78]. Growth inhibitory effects were found in both cell culture and animal models.

A second generation of polyamine analogues are unsymmetrically substituted compounds that display structure-dependent and cell type specific effects on polyamine metabolism [17,79,80]. Another series of polyamine analogues are designated as conformationally restricted, cyclic oligoamines [81]. Some of these agents have limitation on the free rotation of single bonds in otherwise flexible molecules such as spermine or its linear analogues. Oligoamines consist of synthetic octa-, deca-, dodeca- and tetradecamines with longer chains than those of natural polyamines and some of them have conformational restriction. These novel polyamine analogues have shown significant activity against multiple human tumors both in vitro and in vivo [82,83,84,85]. Oligoamines do not highly induce polyamine catabolic enzymes but can still inhibit tumor cell growth and induce apoptosis. Multiple apoptotic mechanisms have been proposed for oligoamine-induced cytotoxic effects, indicating that cell growth inhibition and cell death might be governed by analogue structural specificity effects [82].

It is interesting to note that MCF-7 cells overexpressing Bcl-2 were resistant to paclitaxel but this resistance was overcome by co-treatment of paclitaxel with BE-3-3-3 [86]. Activation of the polyamine catabolic pathway appeared to play a role in inducing cell death by combination therapeutic approach. Polyamine analogues are known to induce reactive oxygen species (ROS) by the activation of polyamine catabolic pathways. Polyamine analogues are known to produce H_2_O_2_ and ROS by the activation of polyamine catabolic pathways and these species play an important role in analogue-induced apoptosis [87]. However, sub-lethal levels of H_2_O_2_ produced by SSAT activation increases susceptibility to skin carcinogenesis [88]. Increased SMO expression is also found in prostate cancer [89]. Polyamine analogues can also interfere with epigenetic modification and expression/re-expression of silenced genes [90]. Taken together, these reports suggest that the polyamine catabolic pathway is a “double-edged sword”, that can participate in carcinogenesis or lead to apoptosis depending on the concentration of H_2_O_2_ and other ROS produced by SMO/SSAT induction [91,92].

## 4. Conclusions

The polyamine metabolic pathway has been an interesting area of research from a molecular biological and drug discovery perspective for half a century. Biosynthetic inhibitors were synthesized and investigated as cancer drug candidates; however, clinical effectiveness was not realized. Polyamine analogues received much attention as a new generation of drug candidates for different forms of cancer, including breast cancer. Several analogues showed excellent anti-cancer efficacy in cell culture and animal models of breast cancer. However, limited clinical studies showed no therapeutic efficacy when bis(ethyl)norspermine was used as a single agent. Combination therapeutic approaches provide new leads to the use of these molecules in breast cancer therapeutics, although clinical studies are yet to be pursued. Several analogues are finding use in nanoparticle strategies [31,93,94]. The metabolism and function of analogues are also being explored using selective deuteration of *N*-alkyl polyamine analogues [95]. These studies provide new insights into the mechanism of action of polyamines and their analogues in cell growth and cell death and point to the importance of additional research to realize the clinical potential of polyamine analogues in breast cancer.
